# The genomic region of the 3′ untranslated region (3′UTR) of *PHO84*, rather than the antisense RNA, promotes gene repression

**DOI:** 10.1093/nar/gkad579

**Published:** 2023-07-18

**Authors:** Youssef A Hegazy, Sara C Cloutier, Sagar M Utturkar, Subhadeep Das, Elizabeth J Tran

**Affiliations:** Department of Biochemistry, Purdue University, BCHM A343, 175 S. University Street, West Lafayette, IN 47907-2063, USA; Department of Biochemistry, Purdue University, BCHM A343, 175 S. University Street, West Lafayette, IN 47907-2063, USA; Purdue University Institute for Cancer Research, Purdue University, Hansen Life Sciences Research Building, Room 141, 201 S. University Street West Lafayette, IN 47907-2064, USA; Department of Biochemistry, Purdue University, BCHM A343, 175 S. University Street, West Lafayette, IN 47907-2063, USA; Department of Biochemistry, Purdue University, BCHM A343, 175 S. University Street, West Lafayette, IN 47907-2063, USA; Purdue University Institute for Cancer Research, Purdue University, Hansen Life Sciences Research Building, Room 141, 201 S. University Street West Lafayette, IN 47907-2064, USA

## Abstract

*PHO84* is a budding yeast gene reported to be negatively regulated by its cognate antisense transcripts both in *cis* and in *trans*. In this study, we performed Transient-transcriptome sequencing (TT-seq) to investigate the correlation of sense/antisense pairs in a *dbp2Δ* strain and found over 700 sense/antisense pairs, including *PHO84*, to be positively correlated, contrasting the prevailing model. To define what mechanism regulates the *PHO84* gene and how this regulation could have been originally attributed to repression by the antisense transcript, we conducted a series of molecular biology and genetics experiments. We now report that the 3′ untranslated region (3′UTR) of *PHO84* plays a repressive role in sense expression, an activity not linked to the antisense transcripts. Moreover, we provide results of a genetic screen for 3′UTR-dependent repression of *PHO84* and show that the vast majority of identified factors are linked to negative regulation. Finally, we show that the *PHO84* promoter and terminator form gene loops which correlate with transcriptional repression, and that the RNA-binding protein, Tho1, increases this looping and the 3′UTR-dependent repression. Our results negate the current model for antisense non-coding transcripts of *PHO84* and suggest that many of these transcripts are byproducts of open chromatin.

## Graphical Abstract

**Graphical Abstract:**
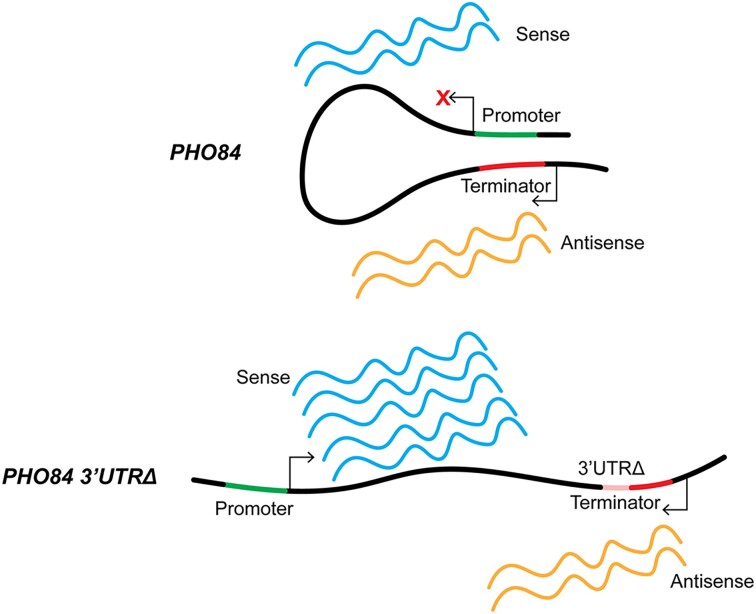

## INTRODUCTION

In both prokaryotes and eukaryotes, transcription occurs on both DNA strands (1–4). The messenger RNA (mRNA) of a gene is regarded as the sense transcript while the non-coding transcript produced from the opposite DNA strand is regarded as antisense transcript (1–4). In budding yeast *Saccharomyces cerevisiae*, 65% of DNA sequences on both strands are composed of non-coding sequences (5,6), while 98% of the human genome is composed of non-coding sequences (7,8).

Long non-coding RNAs (lncRNAs) are a major class of non-coding RNAs that are implicated in gene expression regulation. LncRNAs are RNA polymerase II (RNAPII) products that lack an open reading frame and are longer than 200 nucleotides (9,10). LncRNAs undergo post-transcriptional modifications like 5′ capping and 3′ polyadenylation (11). They can also be spliced, giving rise to various isoforms with the potential for alternative functions (11). LncRNAs regulate gene expression through a variety of different mechanisms. Roles of lncRNAs range from epigenetic to transcriptional and post-transcriptional roles (12–14). For example, the lncRNA GClnc1 was found to favor specific histone modifications by binding, modulating, and coordinating the localization of WDR5 (a component of histone methyltransferase complex) and the histone acetyltransferase KAT2A complexes, thus promoting gastric carcinogenesis (12,15). LncRNAs can recruit transcription factors to their target promoters, as in the case of the lncRNA *MALAT1* which recruits the Sp1 transcription factor to the promoter of *LTBP3*. This leads to transcriptional activation of *LTBP3*, a key gene in multiple myeloma, thus promoting disease progression (13,16). LncRNAs modulate alternative splicing of their target genes by directly interacting with splicing factors, hijacking them from binding their pre-mRNA targets (17,18). They can even act as microRNA (miRNA) sponges sequestering miRNAs in the cytoplasm and preventing them from binding their target mRNAs, thus affecting mRNA stability or translation (13,14,19).

LncRNAs are classified into four main categories based on their genomic locations: (i) intergenic, which are transcribed from intergenic regions and are called long intergenic non-coding RNAs (lincRNAs); (ii) intronic, which are transcribed from introns; (iii) antisense, which are transcribed from the complementary strand of the mRNA-coding strand; (iv) bidirectional, which originate from the same promoter region of the mRNA but from the opposite strand going the opposite direction (20).

In this study, we focus on antisense lncRNAs, referred to as antisense transcripts for simplicity. About 30% of human genes express antisense transcripts that are implicated in gene regulation (20,21). In budding yeast, the percentage of genes with overlapping transcription of sense and antisense transcripts is similar to that of the human genes (this study). Non-coding RNAs produced from antisense transcription are generally unstable (22,23). Therefore, studies investigating non-coding RNA roles in budding yeast have been performed in mutants to stabilize those transcripts (3,22–28). This raises the question of whether those observed roles are innate to those transcripts or if they arise as a consequence of deleting genes encoding regulatory factors.

Dbp2 is a major RNA helicase that belongs to DEAD-box protein family (2,29,30). Studies indicate that DEAD-box proteins exhibit diverse biochemical activities *in vitro*, such as RNA duplex unwinding, RNA folding, and RNP remodeling (31–33). Dbp2 has been linked to transcriptional regulation in budding yeast through various mechanisms. It modulates RNA structure and plays a role in transcriptional termination (30,34). It was also reported to function in transcriptional fidelity and to repress aberrant transcription initiation (35). Published studies from our group provided the first evidence that the galactose (*GAL*) gene cluster-associated lncRNAs (*GAL* lncRNAs) function as transcriptional inducers via R-loop formation, an activity regulated by Dbp2 (2). This direct link between Dbp2 and lncRNAs has prompted us to investigate the global role of Dbp2 on sense/antisense correlation.

*PHO84* is one example of the *S. cerevisiae* genes suggested to have a reciprocal expression pattern of its sense and antisense transcripts. The antisense transcripts of *PHO84* are 2 lncRNAs that have been previously reported to suppress the sense transcription of *PHO84* (3,28). The sense transcript of *PHO84* was reported to be repressed upon deletion of the nuclear exosome component *RRP6* and subsequent stabilization of the antisense transcripts of *PHO84 (*3,26–28).

In this study, we revisited the role of the yeast *PHO84* antisense transcripts and found that the model of antisense-mediated repression does not always hold true. Instead, we report that the 3′UTR of *PHO84* constitutes a regulatory element to *PHO84* sense transcript. Additionally, we provided a list of factors that promote the 3′UTR-dependent regulation of *PHO84*. Our results increase our understanding of transcriptional regulatory networks, and shed light on outstanding questions in the field of RNA biology.

## MATERIALS AND METHODS

### Yeast strain construction

Strains were constructed using standard yeast genetics methods. Primers used for making PCR products used for homologous recombination are listed in Table S4. For *PHO84 3′UTRΔ* strain, the *Delitto Perfetto* technique was used as described before (36) to precisely delete the first 100 bp of the 3′UTR. Primers used for *Delitto Perfetto* technique are listed in Table S5. For the Synthetic Genetic Array (SGA) strains used in this study, a strain carrying *hoΔ::AgSTE3pr-hygR* mutation was obtained from Charles Boone lab and was further mutated to carry can1::LEU2 and either *pho84::HIS3* or *pho84::HIS3 3′UTRΔ* mutations. Strain genotypes and oligos used are listed in Tables S2 and S4.

### Plasmids and cloning

The *EGFP* plasmid was constructed by cloning *EGFP* from pYM28 into p415-ADH yeast expression vector using XbaI, XmaI sites. *EGFP-PHO84 3′UTR* plasmid was constructed by first inserting the first 100 bp of the 3′UTR of *PHO84* downstream of *EGFP* in pYM28 using the BamH1 site and then cloning *EGFP-PHO84 3′UTR* into p415-ADH yeast expression vector using XbaI, XmaI sites. To insert the *PHO84* promoter region into the two plasmids described above, 725 bp upstream of the *PHO84* start codon were used to replace the ADH promoter upstream of *EGFP* using SacI, XbaI sites. Plasmid details are listed in Table S3 and primers used are listed in Table S8. For testing the *trans* effect of the lncRNA, site-directed mutagenesis was used to mutate three binding sites of Pho4. Plasmid details are listed in Table S3 and primers used are listed in Table S10.

### Gene ontology (GO) term analysis

GO term analysis was performed using *Saccharomyces* Genome Database (SGD) Gene Ontology Mapper tool (https://www.yeastgenome.org/goSlimMapper). Functional network analysis was performed using the ClueGo plug-in (Version 2.5.7) of the Cytoscape software (Version 3.5.0) on the 27 positive hits mapped to the GO term ‘transcription by RNA polymerase II’ (GO:0006366). ClueGo analysis settings include checking GO biological process, GO molecular function, and KEGG, with network specificity being set to medium and showing only pathways with pV <0.05.

### Serial dilution spot assay

Log phase cultures of *pho84::HIS3* and *pho84::HIS3 3′UTRΔ* reporter strains were serially diluted with sterile water to make five serial dilutions in a 5× increments. 5 μl aliquots were plated on YPD or on a selective medium lacking histidine and having 25 mM 3-amino-1,2,4-triazole (3-AT) which is a known inhibitor of His3 function. Plates were incubated at 30°C until growth was visible.

### Northern blotting

Northern blotting was performed as described previously (37). gDNA was used as PCR templates for northern blotting probes. Probes labeled with ^32^P-dCTP were generated from PCR templates using the Decaprime II kit according to manufacturer's instructions (Invitrogen). RNA samples were run on an agarose-formaldehyde gel and then transferred to a nylon membrane. After hybridization, Northern blots were quantified by densitometry using ImageQuant TL v7.0 (GE). Transcript levels were determined relative to the *SCR1* Control. Primers used for making Northern probes are listed in Table S6.

### Yeast TT-seq:

Yeast strains transformed with an empty URA vector, pRS426, were grown at 30°C in SD-URA + 2% glucose media to an OD_600_ of 0.5. Cultures were then treated with 4-thiouracil at a final concentration of 0.32 mg/ml for 10 min at 30°C. Cultures were fast cooled in a dry ice/ethanol bath before pellets were harvested by centrifugation and stored at −80°C. RNA was extracted using a hot phenol protocol. RNAs were sonicated to an average size of 1 kb in a Covaris sonicator. 4-Thiouracil-containing RNAs were biotin-labeled using EZ-link biotin (Sigma) at a final concentration of 0.2 mg/ml for 2 h at room temperature. Treated RNAs were extracted twice with chloroform and ethanol precipitated. Biotinylated RNAs were enriched using the μMACS Streptavidin kit (Miltenyi) as per manufacturer's instructions. Enriched RNAs were ribodepleted using a Ribominus yeast/bacteria ribodepletion kit (Thermofisher) and size selected using an RNA Clean and Concentrator-5 kit (Zymo Research). Libraries were prepared using the NEBNext Ultra II Directional RNA Library kit (NEB) as per manufacturer's directions. Sequencing was performed on an Illumina NovaSeq (Novogene).

### Analysis of sense versus anti-sense transcription

Coordinates for predicted non-coding transcripts (CUTs, SUTs and XUTs) (22,23), and coding genes (38,39) in *Saccharomyces cerevisiae* were collected. Genes and non-coding transcripts coordinates were converted into Granges (i.e. an efficient format for storing, manipulating, and aggregating genomic locations data) using the R-package GenomicRanges (v1.38.0) (40). The *join_overlap_inner ()* function from R-package Plyranges (v1.6.10) (41) was applied to determine genes and non-coding transcripts that have overlapping coordinates (a total of 2389 features). Data were further filtered to keep the features that are on opposite strands. These correspond to 2376 non-coding transcripts overlapping with 1669 yeast genes. Data were further filtered to keep the longest non-coding transcript per gene (complete table provided as supplementary table S11). Differential expression was determined (treatment versus control) using coordinates for 1669 non-coding transcripts and yeast genes in three independent datasets. For each dataset, the Pearson correlation between log (fold-change) at gene and non-coding transcripts level was determined using the *chart.Correlation ()* function from the R-package PerformanceAnalytics (v2.0.4) (42). The *chart.Correlation ()* function is equipped to calculate the correlation matrix based on Pearson's product-moment correlation coefficient as well as compute the statistical significance of the correlation results (42), followed by correlation visualizations using the R visualization package ggplot2 (v3.4.2).

### Genetic screen

The *pho84::HIS3* and the *pho84::HIS3 3′UTRΔ* reporters were constructed in a SGA-compatible yeast strain (Strain genotypes are listed in Table S2). The SGA *pho84::HIS3* reporter strain was crossed with an ordered array of ∼5000 strains from Yeast GST-tagged ORF collection (Horizon, YSC4423), each carrying a galactose-inducible overexpression plasmid. After mating, diploids were selected and allowed to sporulate. Haploids carrying the *pho84::HIS3* reporter and an overexpression plasmid were then selected using the mating type-specific reporter (*AgSTE3pr-hygR)*, the plasmid marker, and the *HIS3* auxotrophic marker. After several pinning and selection steps, haploids were finally pinned on three different media: −Ura + 2% galactose, −Ura −His + 40 mM 3AT + 2% galactose and −Ura −His + 40 mM 3AT + 2% glucose. Colony sizes were compared and the ones with growth defect on the −Ura −His + 40 mM 3AT + 2% galactose media only were identified as potential hits. The same steps were repeated after crossing the SGA *pho84::HIS3 3′UTRΔ* strain with an array of the potential positive hits identified from the initial screen. The hits with the galactose-specific growth defect that were completely rescued in the second screen were identified as true hits.

### Chromosome conformation capture (3C)

100-ml cultures of wild-type and *PHO84 3′UTRΔ* strains were grown in SC + glu media to an OD_600_ of ∼0.5 and then crosslinked with 1% formaldehyde and harvested. Cultures of wild-type strains harboring overexpression plasmids were grown in −Ura + glu media to an OD_600_ of ∼0.4, then shifted to −Ura + 2% raffinose for 2 h and then to −Ura + 2% galactose for 5 h and then crosslinked with 1% formaldehyde and harvested. Crosslinked chromatin was isolated by first cryolysing yeast cells and washing the resulting powdered lysate twice with 1× FA lysis buffer [50 mM HEPES·KOH, pH 7.5; 140 mM NaCl; 1 mM EDTA; 1% Triton X-100] and the pellet was resuspended in 500 μl 10 mM Tris·HCl, pH 8.0. SDS was added to 50 μl of the lysate at a final concentration of 0.1% and incubated at 65°C for 10 min to solubilize chromatin. Tubes were then placed immediately on ice for 5 min. SDS was quenched by adding Triton X-100 to a final concentration of 1%. Restriction digestion was performed overnight using 5 μl (50 U) of NlaIII (NEB) in 1× rCutSmart buffer (NEB) and then deactivated by heating at 65°C for 20 min with 10 μl of 10% SDS. Tubes were placed immediately on ice for 5 min and Triton X-100 was added to a final concentration of 1%. The mixture was diluted with water to a total volume of 750 μl. An aliquot of 50 μl was used to perform a digestion check and the ligation step was performed on the remaining 700 μl. PCR using convergent primers (primer 4 & 5 listed in Table S9) was performed after RNase A treatment and phenol/chloroform extraction steps. Digestion was confirmed by the absence of a PCR product on an agarose gel. Ligation was performed by overnight incubation at 16°C using 4.5 μl (1800 U) of T4 DNA ligase (NEB) in 1× T4 DNA ligase buffer (NEB). After incubation, 1 μl (1 μg) of DNase-free RNase A (Ambion) was added and incubated at 37°C for 30 min. SDS was added to a final concentration of 0.1% and 5 μl of Proteinase K (≥800 U/ml, Sigma-Aldrich) was added and the mixture was incubated at 65°C overnight to reverse the crosslinking. Samples were phenol extracted, ethanol precipitated and resuspended in 50 μl nuclease-free water. DNA concentration was determined using Nanodrop. PCR using divergent primers was then performed to detect interactions.

### Strand-specific RT-qPCR

As described previously (2). Primers are listed in Table S7.

## RESULTS

### Sense/antisense pairs show positive correlation

**Figure 1.**
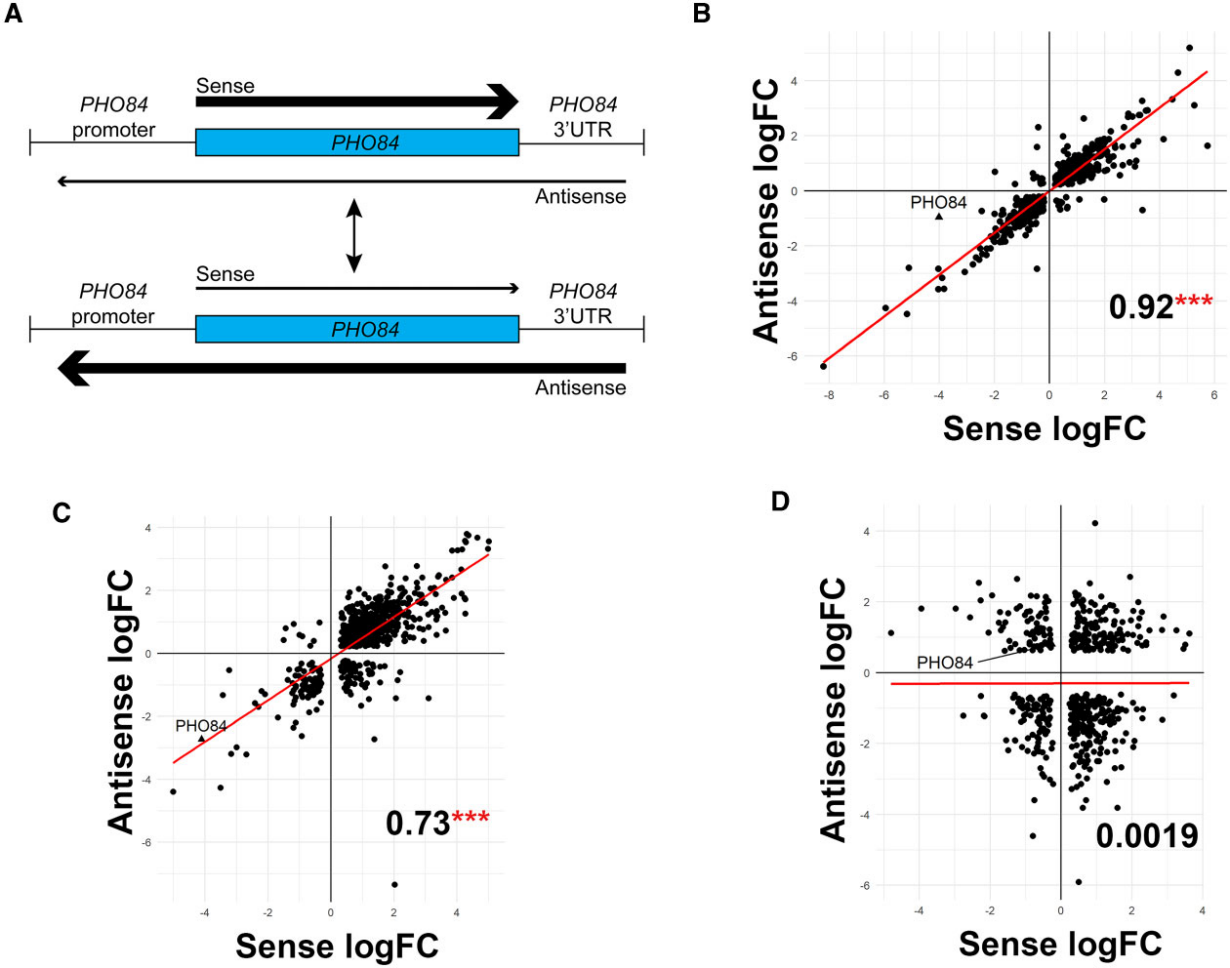
Sense/antisense show positive correlation in wild-type vs. *dbp2Δ* strains. (**A**) Schematic representation of the previously suggested model of sense/antisense negative correlation (3,26–28). (**B**) Correlation plot showing positive correlation between sense and antisense transcription in *dbp2Δ* compared to wild-type. Transient transcriptome sequencing (TT-seq) was performed using three biological replicates of each of *dbp2Δ* and wild-type strains. Genes and non-coding transcripts with overlapping coordinates and located on opposite strands were determined using R-packages. Differential expression analysis was performed on 1669 genes identified as having overlapping sense/antisense transcription. Sense/antisense correlation for the regions that are significant at FDR < 0.25 at gene level is shown. The correlation coefficient and statistical significance denoted by asterisks (*** equivalent to *P*-value < 0.001) are shown in the bottom right quadrant of the plot. (**C**) Correlation plot showing positive correlation between sense and antisense RNA steady-state levels in *dbp2Δ* compared to wild-type. RNA-seq data was obtained from a prior study (30). Differential expression analysis was performed as above. Sense/antisense correlation for the regions that are significant at FDR <0.25 at gene levels is shown. The correlation coefficient and statistical significance denoted by asterisks (*** equivalent to p-value < 0.001) are shown in the bottom right quadrant of the plot. (**D**) Correlation plot showing no correlation between sense and antisense differential expression in *xrn1Δ* compared to wild-type. RNA-seq data was obtained from a prior study (23). Differential expression analysis was performed as above. Sense/antisense correlation for the regions that are significant at FDR <0.25 at gene level are shown. The correlation coefficient is shown in the bottom right quadrant of the plot (no asterisks means non-significant).

The antisense transcripts of *PHO84* have been suggested to silence sense transcription (Figure 1A) (3,26–28). Dbp2, a major DEAD-box RNA helicase in *S. cerevisiae*, was reported to be involved in antisense-dependent gene regulation at the *GAL* gene cluster (2). In this study, we wanted to investigate the effect of *DBP2* on the sense/antisense regulation. More specifically, we wanted to see how the loss of *DBP2* would influence the sense and antisense transcript levels of the genes with overlapping transcription. Transient-transcriptome sequencing (TT-seq), a technique that allows for detection of nascent transcripts (43), was performed in wild-type and *dbp2Δ* strains. This technique has not been previously employed to study the transcriptional impact of antisense RNA. For TT-seq, cultures were treated with 4-thiouracil for a short period of time and nascent transcripts that incorporate the 4-thiouracil were then enriched and sequenced. We identified 1669 gene loci showing overlapping sense and antisense transcripts (Figure 1B–D). We then performed differential gene expression analysis (DE) between wild-type and *dbp2Δ* strains for sense and antisense transcripts. Interestingly, we found a strong positive correlation between sense and antisense transcript levels with a correlation coefficient of 0.92 (Figure 1B). Surprisingly, *PHO84* also showed a sense/antisense positive correlation (Figure 1B), which directly contradicts the current model for antisense-mediated repression of *PHO84* (3,26–28). To determine whether this observation is also observed at steady-state RNA levels, we re-analyzed our prior published *dbp2Δ* RNA-seq dataset (30). Consistent with the TT-seq, we saw a positive correlation in sense/antisense steady-state RNA levels (Figure 1C). This was again true for *PHO84* (Figure 1C).

The *PHO84* antisense transcripts have been proposed to function *in trans*, so we asked if deleting the cytoplasmic decay factor, *XRN1* would influence sense/antisense correlation. Xrn1 is a 5′-3′ RNA exonuclease that is known to regulate a class of non-coding RNAs termed XUTs, and loss of *XRN1* has been suggested to enhance antisense-mediated repression (23,44). Thus, we re-analyzed publicly available RNA-seq data of *xrn1Δ* strains (23). Interestingly, this data showed no global correlation between sense and antisense RNA levels with a correlation coefficient of 0.00 (Figure 1D). Since we see different correlation patterns in different strains, this suggests that it is not the antisense transcripts that regulate the sense transcription, but rather the protein factors that play roles in the regulation of this locus. It is also possible that Xrn1-degraded RNAs that become stable after Xrn1 deletion play a role opposite to that of non-Xrn1-dependent ones.

### 3′UTR deletion of the *PHO84* sense transcript leads to increased sense expression at the *PHO84* locus

**Figure 2.**
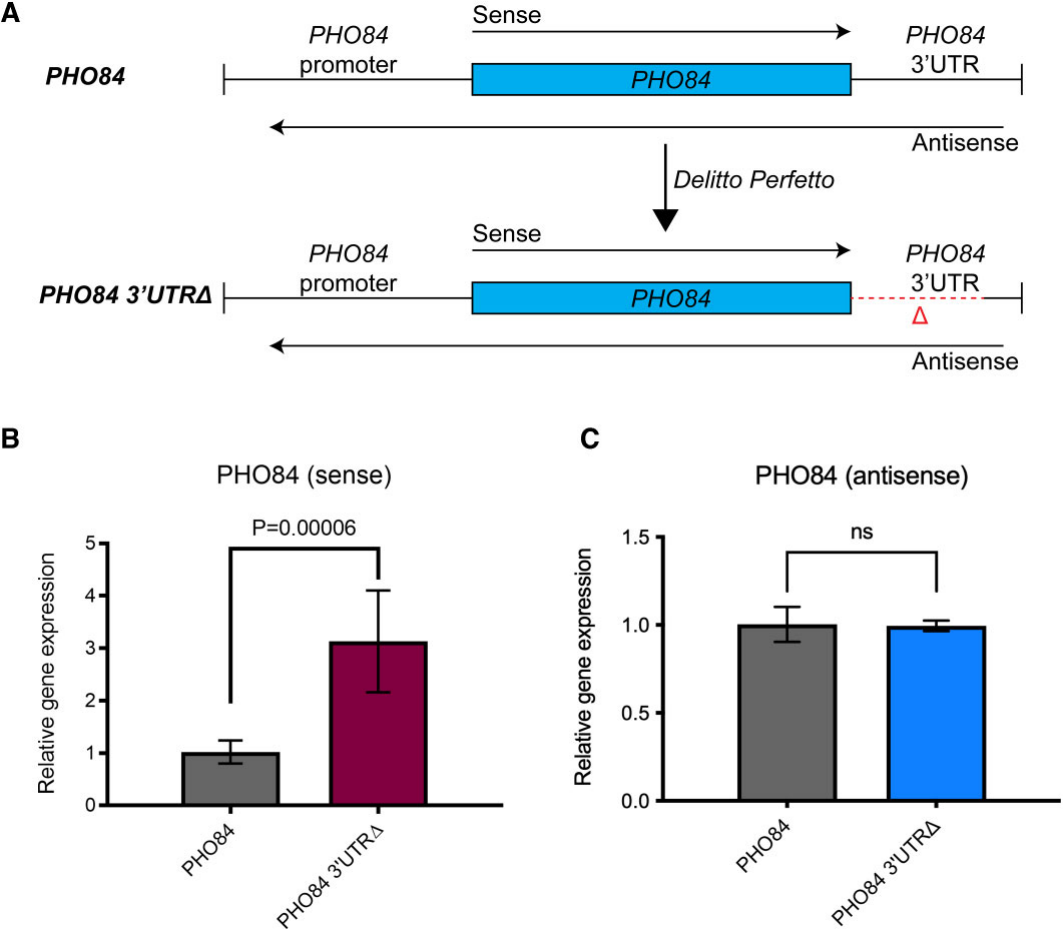
*PHO84* 3′UTR deletion leads to increased sense transcript levels.**(A**) Schematic representation of the wild-type *PHO84* and *PHO84 3′UTRΔ* endogenously-encoded genes. The 3′UTR deletion was constructed by the *Delitto Perfetto* technique (36). (B, C) Strand-specific RT-qPCR (ssRT-qPCR) showing the effect of deletion of the *PHO84* 3′UTR on the sense (**B**) and the antisense transcript levels (**C**). RNA was prepared from 3–8 biological replicates of wild-type *PHO84* and *PHO84 3′UTRΔ* strains grown in SC + glu medium. SsRT-qPCR was conducted to determine the RNA levels of sense and antisense *PHO84*. *ACT1* was used as an internal control. A two-tailed *P* value was calculated using an unpaired *t*-test using GraphPad Prism 9. Error bars represent the standard deviation (s.d.), while *ns* denotes no significance (*P* value > 0.05).

The observation of sense/antisense positive correlation at the *PHO84* gene locus in the *dbp2Δ* strain prompted us to study the regulation of *PHO84* more closely. *PHO84* has been cited as a well-studied example of a gene that is repressed by its cognate antisense transcripts (3,26–28). However, our data showing positive correlation of sense/antisense transcripts suggests that the model of antisense-mediated repression of *PHO84* is not correct. To pinpoint the effect of the antisense transcripts of *PHO84* on sense expression, we deleted 100 bp downstream of the *PHO84* stop codon using the *Delitto Perfetto* technique to remove the previously published *PHO84* antisense promoter site (3) (Figure 2A). We then performed strand-specific reverse transcription qPCR (ssRT-qPCR) for sense and antisense transcripts of *PHO84* in wild-type and *PHO84 3′UTRΔ* strains. Surprisingly, we found that the deletion of the *PHO84* 3′UTR led to a significant 2-fold increase in the sense transcript level without affecting the antisense transcript levels (Figure 2B, C). This suggests that elements within the 3′UTR of *PHO84* are responsible for repression, rather than the antisense transcripts. It also suggests that the promoter of the antisense lncRNA is mis-annotated in the previous study (3).

### SGA screen identified factors promoting 3′UTR-dependent repression in *pho84::HIS3* reporter

**Figure 3.**
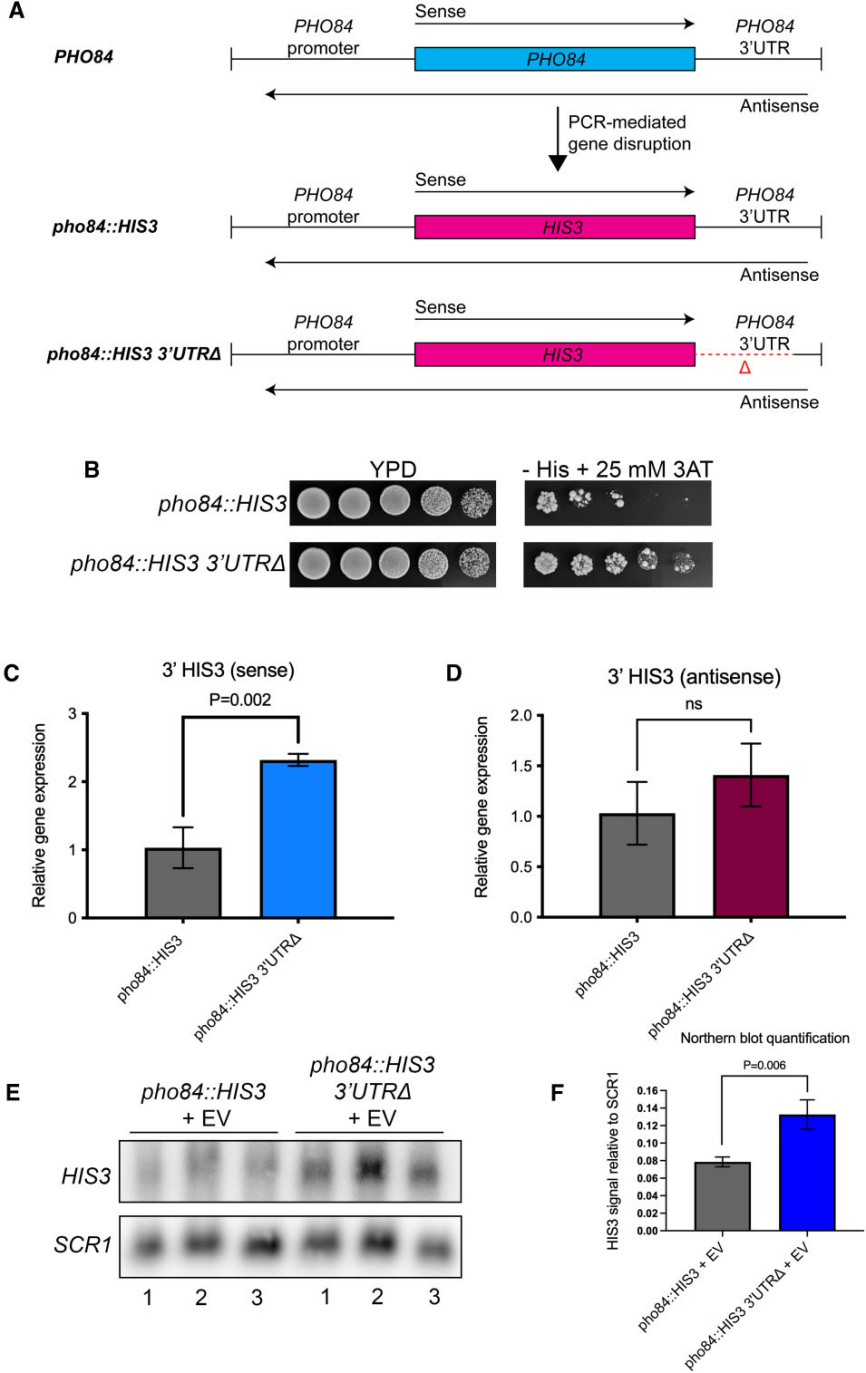
Deletion of the 3′UTR in a *pho84*::*HIS3* reporter leads to increased sense transcript levels.(**A**) Schematic representation of two *pho84::HIS3* reporter strains constructed by replacing the *PHO84* ORF with the *HIS3* ORF using PCR-mediated gene disruption. The deletion of the 3′UTR was constructed by PCR-mediated gene disruption. (**B**) Serial dilution spot assay showing the effect of deleting the 3′UTR of *PHO84* on the growth of *pho84::HIS3* strain on YPD and on selective medium lacking histidine and containing 3-amino-1,2,4-triazole (3-AT). (C, D**)** SsRT-qPCR showing the effect of deletion of the *PHO84* 3′UTR in the *pho84::HIS3* reporter locus on sense (**C**) and antisense transcript levels (**D**).(C, D) RNA was prepared from three biological replicates of each of *pho84::HIS3* and *pho84::HIS3 3′UTRΔ* strains grown in SC + glu medium. SsRT-qPCR was conducted to determine the RNA levels of sense and antisense *pho84::HIS3*. *ACT1* was used as an internal control. A two-tailed *P* value was calculated using an unpaired t-test using GraphPad Prism 9. Error bars represent the s.d. and *ns* denotes no significance (*P* value > 0.05). (E, F) Northern blot of *HIS3* expressed in *pho84::HIS3* and *pho84::HIS3 3′UTRΔ* carrying an empty vector (EV), pRS426 (**E**). Three biological replicates of cultures were grown in a –Ura + glu medium, shifted to –Ura + 2% raffinose for 2 hours, then shifted to –Ura + 2% galactose for 5 hours before harvesting. RNA was prepared and Northern blot was performed. *HIS3* and *SCR1* bands were quantified by densitometry using ImageQuant TL v7.0 (GE) and the intensity of *HIS3* bands relative to those of the loading control, *SCR1*, was plotted (**F**). A two-tailed *P* value was calculated using an unpaired *t*-test using GraphPad Prism 9. Error bars represent the s.d.

To understand how 3′UTR-dependent *PHO84* gene regulation occurs, we constructed a reporter strain for *PHO84* similar to that used in a prior study (28). To this end, we replaced the open reading frame (ORF) of *PHO84* with the open reading frame of the auxotrophic marker *HIS3* (Figure 3A). We also constructed another reporter strain with the first 100 bp of the 3′UTR deleted. When *HIS3* is expressed, cells can synthesize histidine and, therefore, can grow on a media lacking histidine. To confirm the functionality of the reporter, a serial dilution spot assay was performed using the *pho84::HIS3* and the *pho84::HIS3 3′UTRΔ* strains (Figure 3B). This resulted in a weak growth of the *pho84::HIS3* strain on a media lacking histidine and having 3-amino-1,2,4-triazole (3-AT), which is a known inhibitor of His3 function (45). The deletion of the 3′UTR rescued the growth defect seen in the *pho84::HIS3* strain, which confirms the observation that the 3′UTR is repressive to *PHO84*. We then performed ssRT-qPCR and found that the deletion of the *PHO84* 3′UTR leads to a 2-fold increase in the sense transcript level of the *pho84::HIS3* reporter with no significant effect on the antisense transcript levels (Figure 3C, D). To further confirm that *HIS3* expression in the *pho84::HIS3 3′UTRΔ* strain is higher than that of the *pho84::HIS3* strain, a Northern blot was performed. This showed a ∼2-fold increase in *pho84::HIS3* transcript levels upon the deletion of the 3′UTR (Figure 3E, F).

Next, we constructed a plasmid that allows for ectopic expression of the antisense transcripts. A plasmid carrying the *pho84::HIS3* reporter was constructed and the three binding sites of the transcription factor Pho4 were mutated using site-directed mutagenesis to knockout sense transcription from the plasmid (primers listed in Table S10). A serial dilution spot assay (Figure S1) showed that the deletion of the 3′UTR rescues the growth of the *pho84::HIS3* reporter strain on a media lacking histidine, as we showed earlier. The deletion of the nuclear exosome component *RRP6* led to suppression of the sense transcript in the *pho84::HIS3* strain, which was also rescued by the deletion of the 3′UTR (Figure S1). The ectopically expressed antisense transcripts did not have any effect on the growth of the reporter strain even when the *RRP6* was deleted, which also questions the *in trans* model of these antisense transcripts (26) (Figure S1).

**Figure 4.**
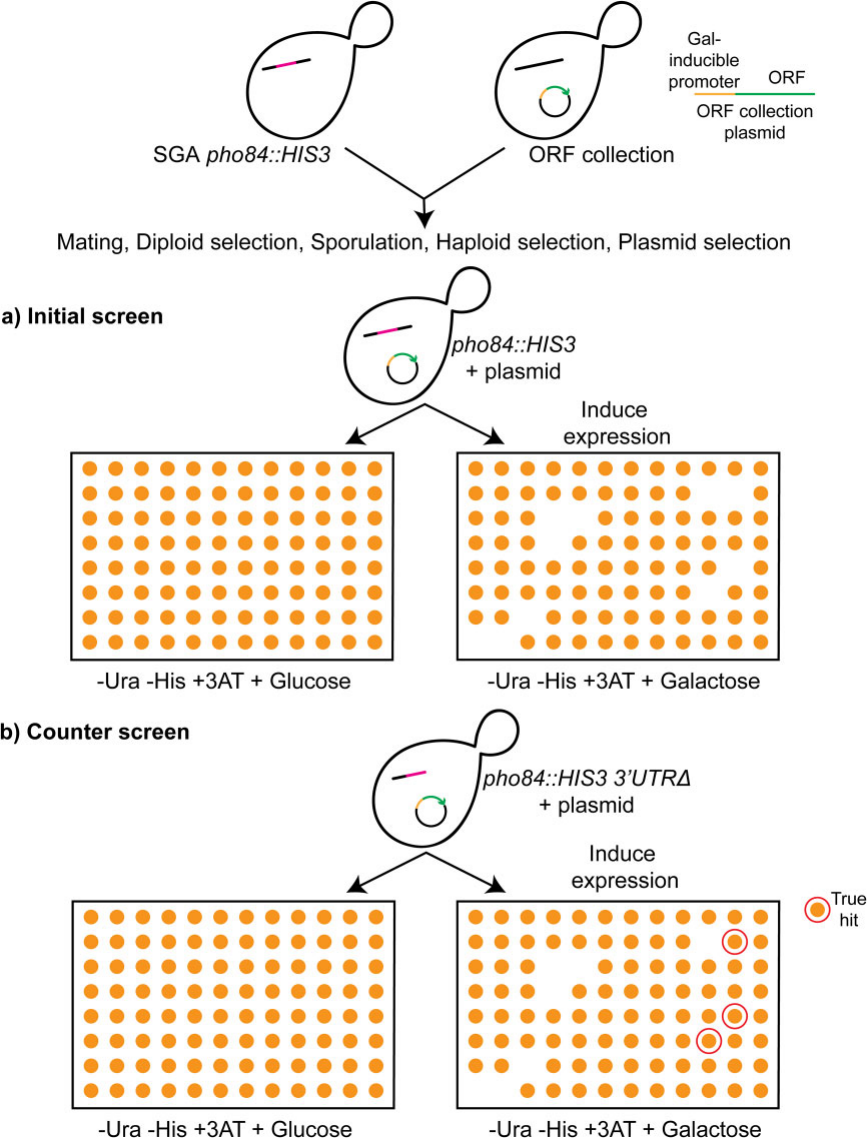
Schematic representation of genetic screen to identify factors that promote 3′UTR-dependent repression in the *pho84*::*HIS3* reporter. The galactose-inducible ORF collection (Horizon, YSC4423) was crossed with the SGA *pho84::HIS3* reporter strain. After selection for diploids, strains were pinned in liquid sporulation medium and allowed to sporulate. Several pinning steps were then performed to select for haploids harboring *pho84::HIS3* reporter and the ORF overexpression plasmids. Strains were then pinned on galactose to induce expression and on glucose for comparison. Strains were scored for galactose-specific growth defects and those were identified as initial hits. A counter screen was then performed by crossing the SGA *pho84::HIS3 3′UTRΔ* strain with an array of the initial positive hits. All the pinning steps described above were repeated and strains were scored for galactose-specific growth defects. The strains whose growth was rescued in the counter screen were identified as true hits.

Next, we employed a synthetic genetic array (SGA) screen to identify factors promoting the 3′UTR-dependent repression (Figure 4). The rationale of the screen was to use the yeast GST-tagged ORF collection (Horizon, YSC4423), which has approximately 5000 strains carrying each ORF under a galactose-inducible promoter in individual strains. These strains were mated with the two reporter strains above, and standard yeast genetics methods were employed to select for haploids encoding the reporter and the overexpression plasmid. We then grew the strains on galactose media (–His + 3AT) to induce overexpression and on non-inducing glucose media for comparison. Strains were also grown on a galactose media that is not selective for *HIS3* to control for any indirect toxic effects of galactose. Plates were then scored for galactose-specific growth defects on the *HIS3*-selective medium. 211 hits were identified to be lethal in the *pho84::HIS3* strain, while having no effect in the *pho84::HIS3 3′UTRΔ* strain (Table S1).

**Figure 5.**
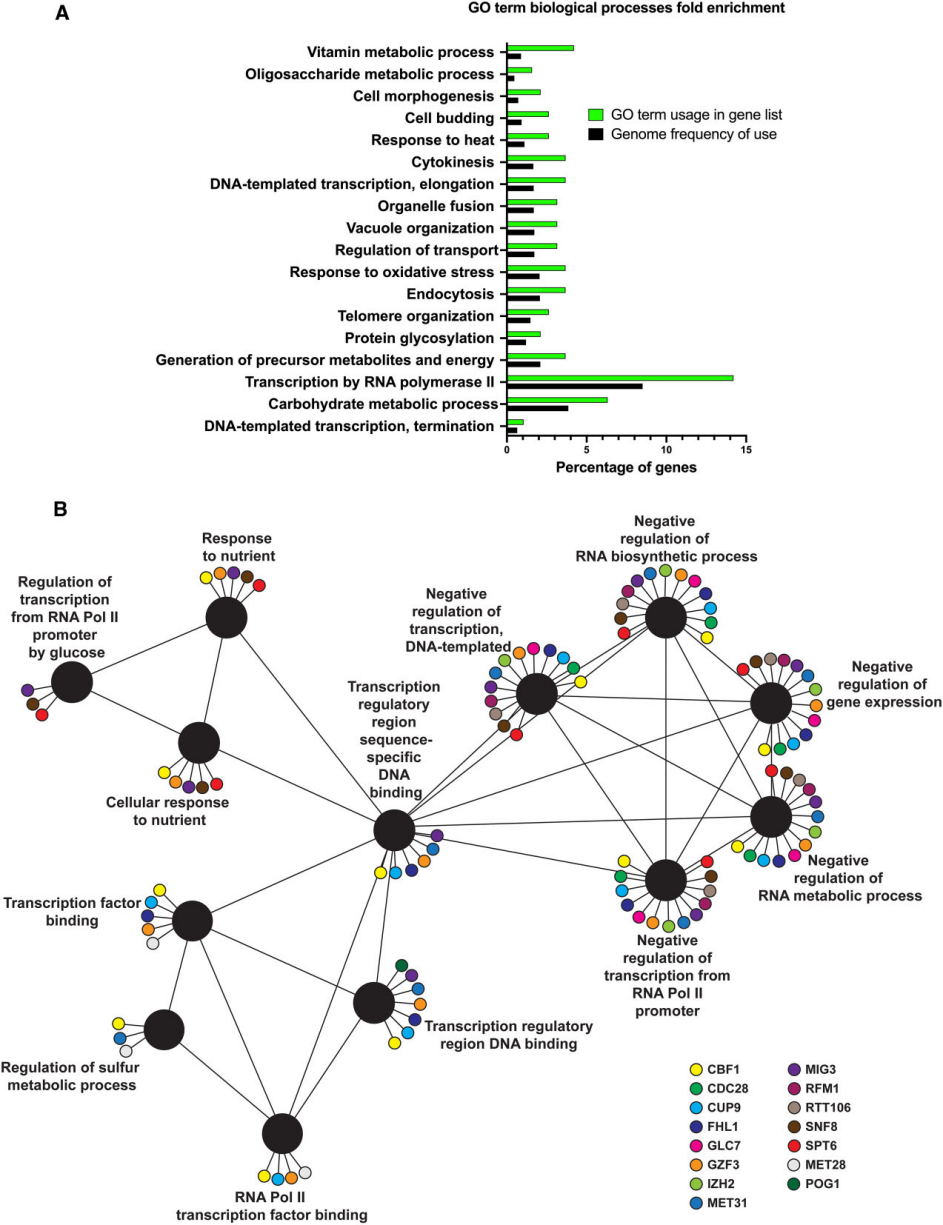
Gene Ontology (GO) term analysis of the genetic screen positive hits showing implication of many factors in transcriptional regulation. (**A**) Bar chart showing GO biological process term enrichment in the gene list obtained from the screen versus the entire yeast genome. GO term analysis was performed using the *Saccharomyces* Genome Database (SGD) Gene Ontology Mapper tool (https://www.yeastgenome.org/goSlimMapper). The percentage of genes mapped to each GO term in the positive hits gene list is shown (green) in comparison to the percentage of genes mapped to each GO term in the entire yeast genome (black). Top highly enriched 18 GO terms are shown. (**B**) Visualization of the biological terms of a subset of the positive hits in a functionally grouped network using the ClueGo plug-in (Version 2.5.7) of the Cytoscape software (Version 3.5.0). 27 positive hits mapped to the GO term ‘transcription by RNA polymerase II’ (GO:0006366), and were then used in the ClueGo network analysis with showing only pathways with pV < 0.05. The black nodes represent the biological terms while the colored small nodes represent the genes mapped to each biological term.

Gene Ontology (GO) term analysis was then performed using the *Saccharomyces* Genome Database (SGD) Gene Ontology Mapper tool (https://www.yeastgenome.org/goSlimMapper). Interestingly, the GO term having the highest number of hits mapped to it (27 out of 211) was ‘transcription by RNA polymerase II (GO:0006366)’ (Figure 5A). Functional network analysis was performed for those 27 hits using the ClueGo plug-in (Version 2.5.7) of the Cytoscape software (Version 3.5.0). This showed that hits are implicated in a number of diverse transcriptional regulatory roles as well as DNA binding (Figure 5B), suggesting a link between the 3′UTR and transcriptional regulation. For instance, Glc7 was one of the hits identified. Glc7 is a phosphatase that is involved in RNA PolII C-termainal domain (CTD) Tyr1 dephosphorylation which is essential for transcription termination (46). In addition, it associates with the cleavage/polyadenylation factor (CPF) (46,47). Tho1, an RNA-binding protein that functions in transcription elongation (48), was also identified from our screen. It binds transcribed chromatin in an RNA-dependent manner (48). Spt6, a well-known histone chaperone, was also identified from the screen. Spt6 was reported to associate with the 3′ ends of transcribed genes and to interact with elongating RNA Pol II (49,50). More recently, Spt6 was found to also play a critical role in controlling the fidelity and specificity of transcription initiation at thousands of genic and intragenic promoters (51).

**Figure 6.**
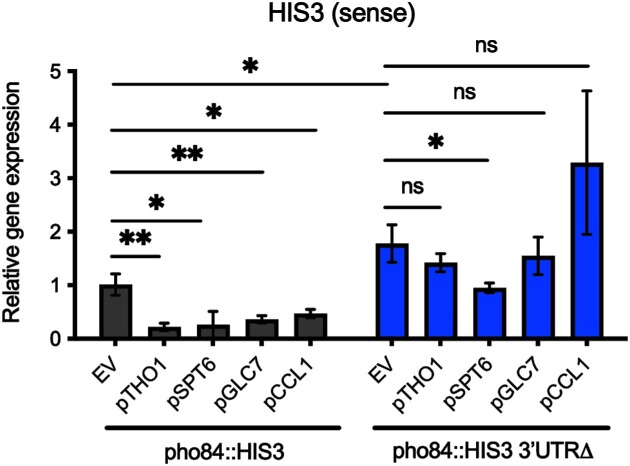
Selected positive hits from the SGA screen show 3′UTR-dependent repression of the *pho84*::*HIS3* reporter. SsRT-qPCR showing the effect of overexpressing four positive hits on the expression of the sense transcript of the *pho84::HIS3* and *pho84::HIS3 3′UTRΔ* reporter strains. RNA was prepared from 3 biological replicates of each strain harboring an empty vector or a plasmid of each ORF under a galactose-inducible promoter. Cultures were grown in -Ura + glu medium and shifted to –Ura + galactose for 17 h. SsRT-qPCR was conducted to determine the RNA levels of sense *pho84::HIS3*. *ACT1* was used as an internal control. A two-tailed *P* value was calculated using an unpaired t-test using GraphPad Prism 9. Asterisk(s) denote significance (*P* value < 0.05), and *ns* denotes no significance (*P* value > 0.05). Error bars represent the s.d.

To further confirm the screen results, we conducted ssRT-qPCR on 4 randomly selected hits from those mapped to the ‘transcription by RNA polymerase II’ GO term. SsRT-qPCR of the sense transcript of the *pho84::HIS3 and pho84::HIS3 3′UTRΔ* reporter strains harboring some selected plasmids showed that the selected hits repress the sense transcript only when the 3′UTR is present (Figure 6), which is consistent with the SGA screen results.

### The 3′UTR is necessary but not sufficient to affect mRNA levels

**Figure 7.**
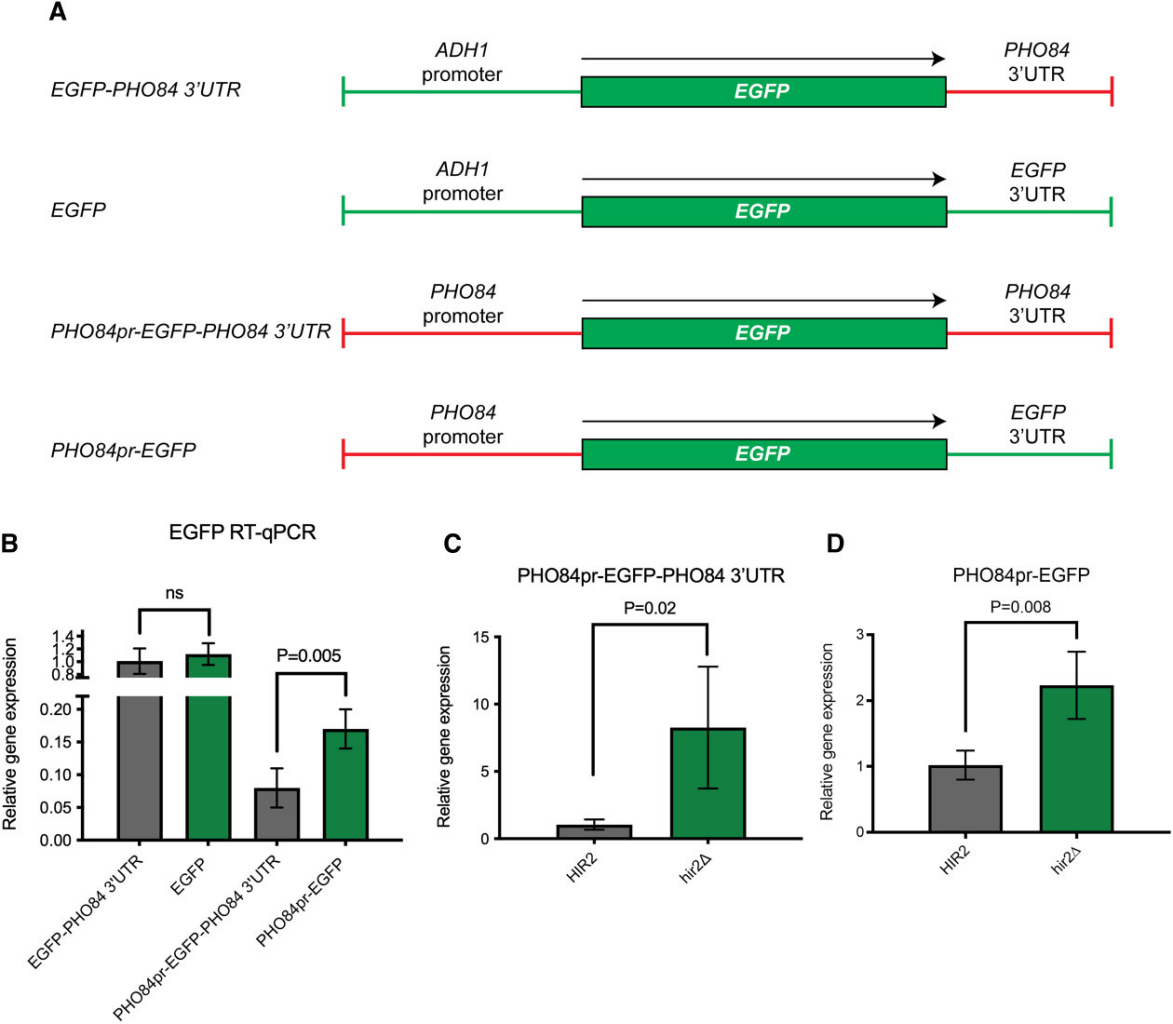
Both the promoter and the 3′UTR are necessary to affect the *PHO84*. (**A**) Schematic illustration of four *EGFP* reporter plasmids constructed to test the effect of the 3′UTR of *PHO84* on the transcript level with and without the presence of the promoter region of *PHO84***. (B**) RT-qPCR performed using a wild-type yeast strain transformed with each of the four constructs. 3–4 biological replicates of each strain were grown in –Leu + glu medium, and shifted to –Leu + 2% raffinose and then shifted to –Leu + galactose then harvested. RNA was prepared and RT-qPCR was performed for *EGFP* using *ACT1* as an internal control. A two-tailed *P* value was calculated using an unpaired t-test using GraphPad Prism 9. Error bars represent the s.d. while *ns* denotes no significance (*P* value > 0.05). (**C**, **D**) RT-qPCR performed using wild-type and *hir2Δ* strains carrying either the *PHO84pr-EGFP-PHO84 3′UTR* or the *PHO84pr-EGFP* plasmid. 3–4 biological replicates of each strain were grown and RT-qPCR was performed for *EGFP* as described above. A two-tailed *P* value was calculated using an unpaired t-test using GraphPad Prism 9. Error bars represent the standard deviation.

To determine how the 3′UTR impacts *PHO84* levels, we constructed a reporter with the 3′UTR of *PHO84* inserted downstream of an *EGFP* ORF on a yeast expression plasmid (Figure 7A). We started by testing the *EGFP-PHO84 3′UTR* construct against the *EGFP* construct that has the wild-type 3′UTR of *EGFP* (Figure 7B). RT-qPCR showed no significant difference in the transcript levels between the two constructs, meaning that the *PHO84* 3′UTR is not sufficient to affect the mRNA levels of *PHO84* (Figure 7B). Next, we replaced the *ADH1* promoter with the *PHO84* promoter region. This led to a significant, 2-fold difference in the mRNA levels between the 3′UTR (+) and the 3′UTR (–) constructs (Figure 7A, B). Thus, the *PHO84* promoter also plays a role in 3′UTR-dependent regulation. This finding also confirms our initial observation that *PHO84* sense transcript regulation is not linked to its antisense transcripts.

A recent study investigating the model of antisense-mediated repression of *PHO84* suggested that the HIR histone chaperone complex, which is involved in *de novo* histone deposition, is involved in this process (28). This study showed that the loss of *HIR2*, one of the four subunits of the HIR histone chaperone complex, leads to an increase in the sense transcript of *PHO84*, an effect that is attenuated upon knocking down the antisense transcripts. In the study, it was suggested that Hir2 and the antisense transcript act together to mediate antisense-mediated repression (28).

We wondered whether *HIR2* deletion would influence the sense transcription in a way that is completely independent of the antisense transcripts. To test this, we conducted an RT-qPCR experiment using wild-type and *hir2Δ* strains carrying the *PHO84pr-EGFP-PHO84 3′UTR* plasmid (Figure 7C). Interestingly, despite the lack of antisense transcription in this construct, we saw an 8-fold increase in the *EGFP* expression in the *hir2Δ* strain, showing that Hir2 plays a role in regulating the sense expression regardless of the presence of the antisense transcripts. This suggests that prior observations may have been the result of impacts on sense transcription rather than a role for the antisense transcripts. Similarly, we also tested the effect of deleting *HIR2* on the *EGFP* expression from the *PHO84pr-EGFP* construct, and we also saw a significant increase in the sense transcript level (Figure 7D). This shows that the role Hir2 plays in regulating the sense expression of *PHO84* is not dependent on the 3′UTR and is solely dependent on the promoter region.

### The *PHO84* gene forms 3′UTR-dependent gene loops

Since the promoter region of *PHO84* is a key player in 3′UTR-dependent gene repression, we wondered whether a physical interaction between the promoter and terminator regions exists. Gene looping has been found to influence transcription both positively and negatively (52–54). The most frequent effect of looping is transcriptional activation (52,53). In these cases, promoter-terminator regions were found to juxtapose each other during active transcription to facilitate RNAPII re-initiation (52,53). Gene looping has also been found to correlate with transcriptional repression at *BRCA1* gene, in which looping is thought to reduce promoter accessibility (55).

**Figure 8.**
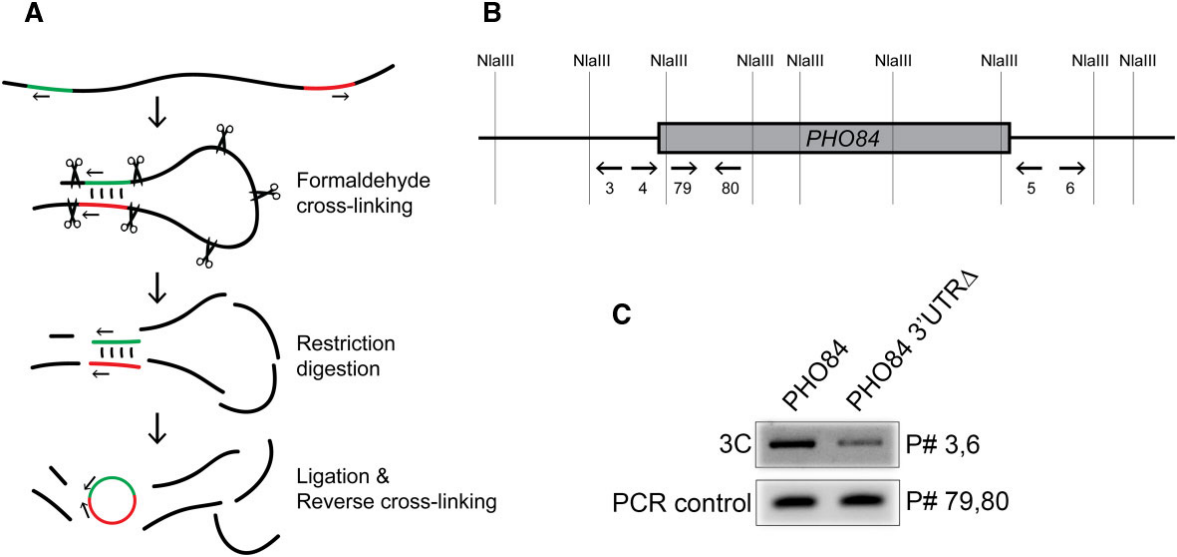
Chromosome conformation capture (3C) reveals interaction between promoter and terminator regions of *PHO84*. (**A**) Schematic representation of the chromosome conformation capture (3C) technique. Cultures were crosslinked with formaldehyde, and then chromatin was isolated and digested with a restriction enzyme. Ligation followed by reverse-crosslinking were then performed. Small arrows represent divergent primers that become convergent after ligation. Green and red denote interacting DNA regions. (**B**) Schematic representation of *PHO84* showing representative NlaIII restriction sites and the location and direction of the primers used below (oligo sequences are listed in table S9). Primers 4 & 5 were used to check for efficient digestion (see Materials and Methods). (**C**) PCR-agarose gel electrophoresis of the ligation products of *PHO84* & *PHO84 3′UTRΔ* strains. PCR was performed using divergent (3 & 6) and convergent (79 & 80) primers. PCR products appearing using divergent primers indicates interaction. PCR using convergent primers was used as a loading control.

**Figure 9.**
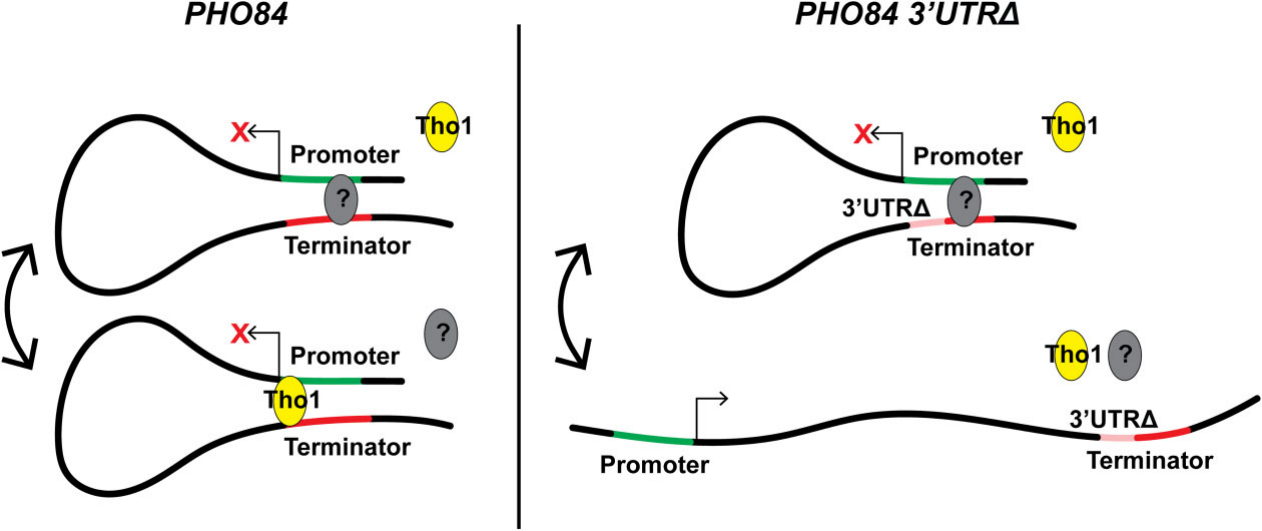
Proposed model for the 3′UTR-dependent repression at *PHO84* locus. Promoter-terminator region interaction at *PHO84* locus is thought to be promoted by transcription factors, resulting in repression. Those factors can be any known factor that promotes gene looping, which could be any of the identified hits from the genetic screen performed in this study. Tho1 was identified in this study to promote gene looping and to cause 3′UTR-dependent repression. When the 3′UTR is deleted, some of those factors can no longer bind, resulting in less frequent interaction between the promoter and terminator regions leading to transcriptionally active state. Other factors may still bind any region downstream of the 3′UTR resulting in looping that is not dependent on the 3′UTR.

To test if the promoter and terminator regions of *PHO84* interact, we conducted a chromosome conformation capture (3C) assay using the endogenous *PHO84* gene in wild-type and *PHO84 3′UTRΔ* strains *(*Figure 8A). PCR using divergent primers was done using *PHO84* and *PHO84 3′UTRΔ* strains. It showed gene looping between the promoter and terminator regions of *PHO84*, as evidenced by obtaining a PCR product using divergent primers (Figure 8B, C). It also shows that this interaction is less frequent when the 3′UTR is deleted (Figure 8B-C); band intensity from the *3′UTRΔ* strain was 58% of that of the wild-type (Figure 8C). This indicates that the promoter and terminator regions of *PHO84* interact and this interaction might play a role in the 3′UTR-dependent repression seen in the *PHO84* gene (Figure 9).

We then sought to test the effect of the positive hits identified from the SGA screen on the *PHO84* gene looping. We used the 4 selected hits previously confirmed to produce 3′UTR-dependent (Figure 6). Interestingly, we found that the overexpression of *THO1* lead to a significant increase in the *PHO84* gene looping (Figure S2), which again links repression to increased looping frequency. However, no significant change in looping frequency was seen with the other three hits tested which suggests that their effect on *PHO84* expression is achieved via alternative mechanisms.

## DISCUSSION

Since 2007, the *PHO84* gene has been cited as a model for antisense-mediated repression (3,28). In line with this, the deletion of the nuclear exosome component, *RRP6*, was found to stabilize the antisense transcripts of *PHO84* and repress the sense transcription (3). It was shown that the antisense transcripts silence sense transcription via recruiting the histone deacetylase Hda1, resulting in local chromatin compaction (3). This activity of the antisense transcripts is only observed when they are stabilized by the deletion of *RRP6*. A more recent study suggested that HIR histone chaperone complex is required for the antisense transcripts to silence sense transcription (28).

Most studies of antisense transcripts in *S. cerevisiae* have been conducted in mutant strains where antisense transcripts can be stabilized and/or extended (3,22,23). This is a caveat for characterizing the influence of those non-coding transcripts on the overlapping mRNAs, because the loss of those factors that regulate antisense transcripts might have either direct or indirect effect on the sense transcription itself.

Recent studies have also employed loss-of-function approaches to study the influence of antisense transcripts. RNA interference (RNAi) using oligonucleotides that target antisense transcripts and strand-specific CRISPR interference (CRISPRi) have been among the main approaches for knocking down antisense transcripts (56,57). However, these techniques suffer from limitations such as off-target effects and incomplete strand specificity (56,57). Off-target effects have been a common issue with CRISPR/Cas9 techniques, and lack of strand specificity could be because the physical presence of a catalytically-dead Cas9 that would interfere with the transcriptional machinery and/or chromatin structure at the opposite strand (56). In addition, almost all studies to date investigating the roles of antisense transcripts in *S. cerevisiae* have employed techniques that look into steady-state levels of RNA (22,23). This may not give an accurate estimation of transcriptional activity, as steady-state levels depend both on transcription and decay.

Taking *PHO84* as an example, the fact that sense/antisense pairs were found to be positively correlated suggests that the antisense-mediated repression model is not valid, and that other factors might be contributing, either directly or indirectly. Additionally, the previously proposed activity of the *PHO84* antisense transcripts was only seen when cells were grown in minimal growth conditions after chronological aging or when *RRP6* is deleted (3), raising concerns of whether these antisense transcripts have physiological roles in normal conditions. Here, we provide the first evidence that the 3′UTR of *PHO84*, together with its promoter region, regulates the sense transcript levels of *PHO84*, and that this regulation is independent on the antisense transcripts in healthy, wild-type cells. Additionally, we provide the first evidence that the *PHO84* locus forms gene loops, and that interaction between the promoter and terminator regions is modulated by the 3′UTR, suggesting that the looping at this locus is repressive. Our data shows that the 3′UTR is not required for the looping to occur, however its deletion significantly lowers the interaction frequency. With the data in hand that shows that Tho1, the RNA-binding protein that is known to bind transcribed chromatin (48), increases the looping and 3′UTR-dependent repression, it's probable that some protein factors bind either the promoter region of *PHO84* or the terminator region including the 3′UTR. It is possible that these factors interact with each other to help bring the promoter and terminator regions in close proximity and this in turn reduces promoter accessibility. This may explain why the loss of the 3′UTR does not completely abolish the looping. Further mechanistic studies are needed to decipher the link between the *PHO84* gene looping and its repression, to investigate whether the looping is repressive *per se*, or it's the *trans*-acting factors, such as Tho1, that bind the 3′UTR interfering with transcription efficiency.

Antisense RNAs are ubiquitous in nature (1). We suggest that research focused on transcriptional roles for antisense transcripts use methods that measure transcription directly rather than the steady-state RNA levels. Moreover, we suggest that caution should be used when determining a role for antisense transcripts solely from data using mutant strains. Approaches investigating the transcriptional roles of antisense RNAs in wild-type cells rather than mutants are immensely needed to avoid experimental bias arising from indirect effects.

Many factors known to be involved in transcriptional regulation were identified in our SGA screen and shown to regulate transcript levels in a 3′UTR-dependent manner. More studies are needed to better characterize the mechanism of these factors which can lead to a better understanding of the role of 3′UTRs in transcriptional regulation. Future studies using a combination of TT-seq and comparison with ChIP-seq may provide clarification of the mechanisms in play. Regardless, our studies provide evidence that the lncRNA-mediated repression model at the PHO84 locus, and likely many more gene loci, is incorrect. Future studies are necessary to understand the role(s) between transcriptional regulation and promoter-terminator looping.

## Supplementary Material

gkad579_Supplemental_Files

## Data Availability

The data reported in this study is available under the GEO accession number: GSE227252.
