# Supplementary material for: The genomic region of the 3′ untranslated region (3′UTR) of PHO84, rather than the antisense RNA, promotes gene repression
Source: Nucleic Acids Res. 2023 Jul 18;51(15):7900–13. doi: 10.1093/nar/gkad579 (PMC10450162; doi:10.1093/nar/gkad579)
Supplement: gkad579_Supplemental_Files [file gkad579_supplemental_files.zip › Supplementary material_Hegazy et al_Updated.pdf]

SUPPLEMENTARY FIGURE 1

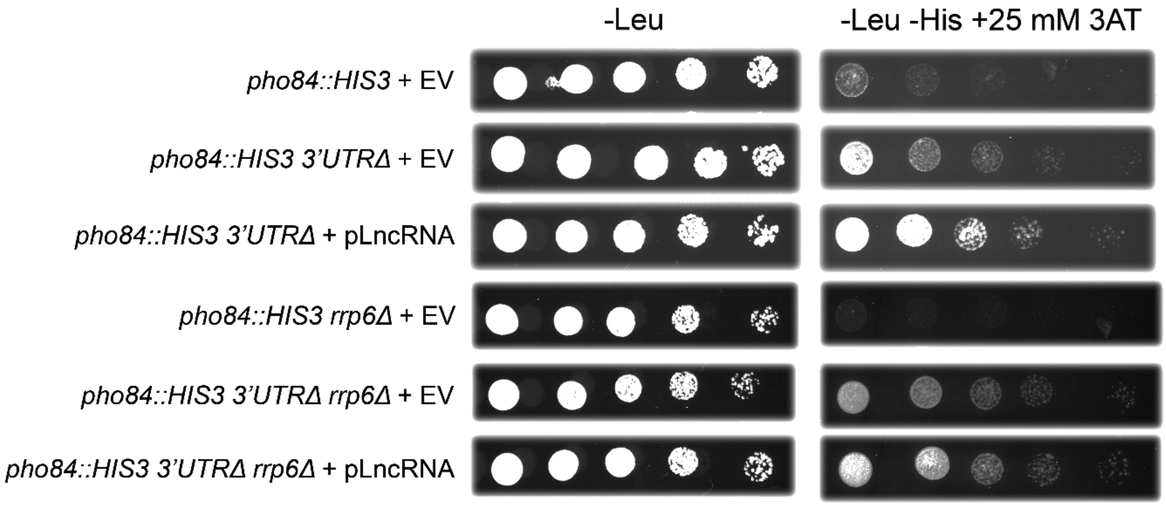

Figure S1. Ectopic expression of the antisense transcripts does not lead to repression.
Serial dilution spot assay showing the effect of overexpressing the antisense transcripts (lncRNA) of *PHO84* on the growth of *pho84::HIS3* strain on -leucine medium and on selective medium lacking leucine and histidine and containing 3-amino-1,2,4-triazole (3-AT). EV stands for empty vector. pLncRNA is a plasmid expressing the antisense transcripts of *PHO84*.

SUPPLEMENTARY FIGURE 2

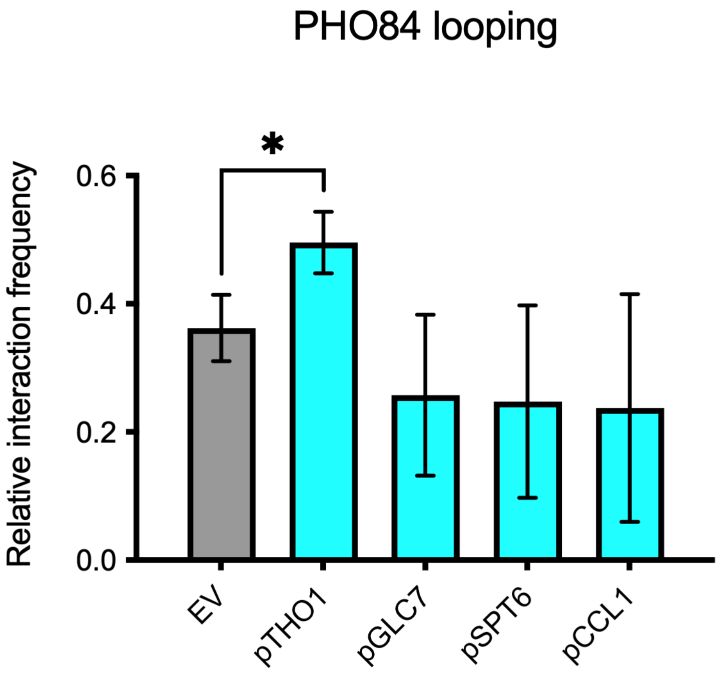

Figure S2. 3C experiment showing that *THO1* overexpression significantly increases promoter-terminator interaction at *PHO84* locus.
Quantification of agarose gel bands of the 3C experiment PCR products using divergent primers (3 & 6) relative to the PCR products of the convergent primers (79 & 80) used as a loading control. 3C was performed on *PHO84* strain harboring either an empty vector, *pTHO1, pGLC7, pSPT6, or pCCL1* plasmid. 3 biological replicates of each strain were used. Strains were grown in -Ura + glu medium until the OD600 of 0.45, then shifted to -Ura + gal medium for 17 hours. A two-tailed P value was calculated using an unpaired t-test using GraphPad Prism 9. Error bars represent the s.d., statistical significance is denoted by asterisks.

TABLE S1

Biological process GO term	GO ID	Genes Annotated to the GO Term			GO Term Usage in Gene List	Genome Frequency of Use	Fold enrichment
Transcription by RNA polymerase II	GO:0006366	*CDC28*<br>*MIG3*<br>*THO1*<br>*GLC7*<br>*SPT6*<br>*POG1*<br>*RFM1*<br>*SNF8*<br>*TAF3*	*MET28*<br>*GZF3*<br>*CBF1*<br>*PHD1*<br>*RSC4*<br>*CGI121*<br>*FHL1*<br>*SGV1*<br>*RPO26*	*ARP9*<br>*MSS11*<br>*RTT106*<br>*IZH2*<br>*RPB11*<br>*INO4*<br>*MET31*<br>*CUP9*<br>*CCL1*	27 of 190 genes, 14.21%	548 of 6439 annotated genes, 8.51%	1.67
Carbohydrate metabolic process	GO:0005975	*UBP14*<br>*CDC28*<br>*GPD1*<br>*RKI1*	*GLC7*<br>*CWH41*<br>*DDP1*<br>*PCK1*	*GSY2*<br>*ERR3*<br>*GCY1*<br>*SUN4*	12 of 190 genes, 6.32%	248 of 6439 annotated genes, 3.85%	1.64
Vitamin metabolic process	GO:0006766	*RIB3*<br>*SNZ3*<br>*RIB4*	*FOL2*<br>*THI11*<br>*RKI1*	*ABZ1*<br>*BIO3*	8 of 190 genes, 4.21%	57 of 6439 annotated genes, 0.89%	4.73
Cytokinesis	GO:0000910	*EDE1*<br>*CDC28*<br>*SUN4*	*GLC7*<br>*MSB2*	*VRP1*<br>*IQG1*	7 of 190 genes, 3.68%	107 of 6439 annotated genes, 1.66%	2.22
DNA-templated transcription, elongation	GO:0006354	*THO1*<br>*RSC4*<br>*SGV1*	*RPA34*<br>*RTT106*	*ARP9*<br>*SPT6*	7 of 190 genes, 3.68%	108 of 6439 annotated genes, 1.68%	2.19
Response to oxidative stress	GO:0006979	*CMK1*<br>*ASK10*<br>*MRD1*	*YHB1*<br>*HYR1*	*NCE103*<br>*GCY1*	7 of 190 genes, 3.68%	132 of 6439 annotated genes, 2.05%	1.8
Endocytosis	GO:0006897	*EDE1*<br>*GLC7*<br>*SLG1*	*PIL1*<br>*CLC1*	*VRP1*<br>*SNC2*	7 of 190 genes, 3.68%	133 of 6439 annotated genes, 2.07%	1.78
Generation of precursor metabolites and energy	GO:0006091	*CDC28*<br>*GLC7*<br>*TKL1*	*GSY2*<br>*ERR3*	*RKI1*<br>*ATF1*	7 of 190 genes, 3.68%	135 of 6439 annotated genes, 2.10%	1.75
Organelle fusion	GO:0048284	*CLN3*<br>*GYP7*	*IVY1*<br>*ERR3*	*STT4*<br>*SNC2*	6 of 190 genes, 3.16%	109 of 6439 annotated genes, 1.69%	1.87
Vacuole organization	GO:0007033	*CLN3*<br>*GYP7*	*IVY1*<br>*YVH1*	*ENV11*<br>*ERR3*	6 of 190 genes, 3.16%	111 of 6439 annotated genes, 1.72%	1.84
Regulation of transport	GO:0051049	*GLC7*<br>*ASK10*	*CLC1*<br>*SEC12*	*RHO3*<br>*SLG1*	6 of 190 genes, 3.16%	111 of 6439 annotated genes, 1.72%	1.84
Cell budding	GO:0007114	*CDC28*<br>*KIC1*	*KCC4*<br>*VRP1*	*GLC7*	5 of 190 genes, 2.63%	59 of 6439 annotated genes, 0.92%	2.86
Response to heat	GO:0009408	*GLC7*<br>*SGT2*	*PIL1*<br>*SLG1*	*WSC3*	5 of 190 genes, 2.63%	71 of 6439 annotated genes, 1.10%	2.39
Telomere organization	GO:0032200	*CDC28*<br>*OGG1*	*GLC7*<br>*IES4*	*CGI121*	5 of 190 genes, 2.63%	95 of 6439 annotated genes, 1.48%	1.78
Cell morphogenesis	GO:0000902	*KCC4*<br>*CIN2*	*GLC7*	*RHO3*	4 of 190 genes, 2.11%	46 of 6439 annotated genes, 0.71%	2.97
Protein glycosylation	GO:0006486	*ANP1*<br>*ALG5*	*CWH41*	*ALG11*	4 of 190 genes, 2.11%	77 of 6439 annotated genes, 1.20%	1.76
Oligosaccharide metabolic process	GO:0009311	*CDC28*	*CWH41*	*YMR196W*	3 of 190 genes, 1.58%	30 of 6439 annotated genes, 0.47%	3.36
DNA-templated transcription, termination	GO:0006353	*SPT6*	*RPB11*		2 of 190 genes, 1.05%	42 of 6439 annotated genes, 0.65%	1.62

Table S1: Biological process Gene Ontology (GO) term analysis results of the genetic screen positive hits.

GO term analysis was performed using the Gene Ontology Mapper tool on the Saccharomyces Genome Database (SGD) website (https://www.yeastgenome.org/goSlimMapper). Terms were ranked by the percentage of the GO term usage in the gene list.

TABLE S2 List of strains

Name	Lab code	Genotype
Wild-type	OPY1	*MATa his3Δ1 leu2Δ0 met15Δ0 ura3Δ0* (BY4741)
dbp2Δ	BTY116	*MATalpha dbp2::KanR ura3Δ0 leu2Δ0 his3Δ0 TRP1 MET LYS*
PHO84	OPY1	*MATa his3Δ1 leu2Δ0 met15Δ0 ura3Δ0* (BY4741)
PHO84 3'UTRΔ	BTY509	*MATa his3Δ1 leu2Δ0 met15Δ0 ura3Δ0 PHO84 3'UTRΔ*
pho84::HIS3	BTY481	*MATa his3Δ1 leu2Δ0 met15Δ0 ura3Δ0 pho84::HIS3*
pho84::HIS3 3'UTRΔ	BTY482	*MATa his3Δ1 leu2Δ0 met15Δ0 ura3Δ0 pho84::HIS3 3'UTRΔ*
SGA *pho84::HIS3*	BTY500	*MATalpha his3Δ1 leu2Δ0 ura3Δ0 hoΔ::AgSTE3pr-hygR can1::LEU2 pho84::HIS3*
SGA *pho84::HIS3 3'UTRΔ*	BTY501	*MATalpha his3Δ1 leu2Δ0 ura3Δ0 hoΔ::AgSTE3pr-hygR can1::LEU2 pho84::HIS3 3'UTRΔ*

TABLE S3 List of plasmids

Name	Lab code	Description
pRS426	OPP16	*URA*+ empty vector
EGFP-PHO84 3'UTR	BTP147	*p415-ADH EGFP-PHO84 3'UTR*
EGFP	BTP148	*p415-ADH EGFP*
PHO84pr-EGFP-PHO84 3'UTR	BTP152	*p415-ADH PHO84 promoter-EGFP-PHO84 3'UTR*
PHO84pr-EGFP	BTP153	*p415-ADH PHO84 promoter-EGFP*
pRS315	OPP3	*LEU*+ empty vector
pLncRNA	BTP116	*pRS315 pho84::HIS3 Pho4p-BSsΔ*

TABLE S4 Oligos for homologous recombination

Name	Sequence	Lab #
dbp2::KanMX F	CAACAACCTGTAACAGAATTAAGCACTATTAAGGCAAATTTAGAGCAAATATGCAGCTGAAGCTTCGTACGC	22
dbp2::KanMX R	GCAGTCAACTTATATAATTATTATTAATAGAGATGAATGAATTGAATCACTTTGGCATAGGCGACTAGTGGATCTG	23
pho84::HIS3 F	TTCCTCATCTCGTAGATCACCAGGGCACACAACAAACAAAACTCCACGAATACAATCCAAATGACAGAGCAGAAAGCCCT	758
pho84::HIS3 R	ATAAAAATGTTTTTGTATTATTTGTTCTAGTTTACAAGTTTTAGTGCATCTTTGAGGCTTCTACATAAGAACACCTTTGG	759
pho84::HIS3 3'UTRΔ F	TTCCTCATCTCGTAGATCACCAGGGCACACAACAAACAAAACTCCACGAATACAATCCAAATGACAGAGCAGAAAGCCCT	758
pho84::HIS3 3'UTRΔ R	AGATGTGAGGAAATAATGAAATTAAAAGAAATTATCGAATAAATATGTAACCTGACAGTACTACATAAGAACACCTTTGG	809
can1::LEU2 F	TTTCAGAGTTCTTCAGACTTCTTAACTCCTGTAAAAACAAAAAAAAAAAAAGGCATAGCACCTCGAGGAGAACTTCTAGTATA	1386
can1::LEU2 R	TATGAGGGTGAGAATGCGAAATGGCGTGGAAATGTGATCAAAGGTAATAAAACGTCATATAGATTGTACTGAGAGTGCACCAT	1387

TABLE S5 Oligos for *Delitto Perfetto* technique

Name	Sequence	Lab #
PHO84::CORE F	GATGCACTAAAACTTGTAAACTAGAACAAATAATACAAAAGAGCTCGTTTTCGACACTGG	1539
PHO84::CORE R	AGATTATGTAAGGGGTTTGATAATAAGTTTATAAAAATGTTCCTTACCATTAAGTTGATC	1540
Oligo 1 for dsDNA used for HR	TGAATCTTCCAGCCCATCTCAACTTCAACATGAAGCATAATCAGGTTACATATTTATTCGATAATTTCTTTTAATTTCAT	1543
Oligo 2 for dsDNA used for HR	ATGAAATTAAAAGAAATTATCGAATAAATATGTAACCTGATTATGCTTCATGTTGAAGTTGAGATGGGCTGGAAGATTCA	1544

TABLE S6 Oligos for Northern probes

Name	Sequence	Lab #
HIS3 F	ATGACAGAGCAGAAAGCCCTAGTAAAG	541
HIS3 F	CTACATAAGAACACCTTTGGTGGAGG	542
SCR1 F	GGATACGTTGAGAATTCTGGCCGAGG	245
SCR1 R	AATGTGCGAGTAAATCCTGATGGCACC	263

TABLE S7 Oligos for RT-qPCR

Name	Sequence	Lab #
PHO84 F	TGGAGTCCATCGATGACGAA	79
PHO84 R	CACCAGCAATGGAGATGGTCTT	80
HIS3 F	GCCTTTGGATGAGGCACTTTC	822
HIS3 R	GCGTACGGCCTGTTCGAA	823
EGFP F	GTCCGCCCTGAGCAAAGA	1589
EGFP R	TCCAGCAGGACCATGTGATC	1590
ACT1 F	TGGATTCCGGTGATGGTGTT	77
ACT1 R	TCAAAATGGCGTGAGGTAGAGA	78

TABLE S8 Oligos for cloning

Name	Sequence	Lab #
For inserting PHO84 3'UTR in pYM28 F	CGAGGATCCAAGCCTCAAAGATGCACTAAAACT TG	1546
For inserting PHO84 3'UTR in pYM28 R	CGAGGATCCCAGTATTTATAGATTATGTAAGGG GTTTGAT	1547
For inserting EGFP-PHO84 3'UTR in p415-ADH F	GGTATCTAGAATGGTGAGCAAGGGCGAGGAGC T	1570
For inserting EGFP-PHO84 3'UTR in p415-ADH R	CGTGCCCGGGCAGTATTTATAGATTATGTAAGG GGTTTGATA	1571
For inserting EGFP in p415-ADH F	GGTATCTAGAATGGTGAGCAAGGGCGAGGAGC T	1570
For inserting EGFP in p415-ADH R	CGTGCCCGGGTTACTTGTACAGCTCGTCCATGC CG	1580
For inserting PHO84 promoter in p415-ADH EGFP F	GTAGAGCTCCAGCCGCTCGTTTATCAACCG	1591
For inserting PHO84 promoter in p415-ADH EGFP R	GTATCTAGATTGGATTGTATTCGTGGAGTTTTGT TT	1592

TABLE S9 Oligos for 3C

Name	Sequence	Lab #	Position from the start codon
3	TGGATCTAAGTTGGCATTACC	1624	-172 to -152
4	TCCTCATCTCGTAGATCACCA	1625	-59 to -39
5	AACAGGATGGCAGAGAGATG	1626	+1914 to +1933
6	ATTGGTCACCATCTGGTTGAT	1627	+2239 to +2259
79	TGGAGTCCATCGATGACGAA	79	+148 to +167
80	CACCAGCAATGGAGATGGTCTT	80	+189 to +210

TABLE S10 Oligos for site-directed mutagenesis

Name	Sequence	Lab #
1st Pho4 binding site mutation F	GGAATAAAAAAGTGTCGCTTGATAAAAATC	1169
1st Pho4 binding site mutation R	CGGGTAGTGATTTTTATCAAGCGACACTTT	1170
2nd Pho4 binding site mutation F	GATGCCAATTTAATAGTTCCGCTTGGACGTG	993
2nd Pho4 binding site mutation R	TGCTGGAAATAACACGTCCAAGCGGAACTA	1151
3rd Pho4 binding site mutation F	ATTAGCTAGATTAAACCCGTGCGTATTA	1175
3rd Pho4 binding site mutation R	TTAATGAGTAATACGCACGGGTTTAATC	1176

Table S11: Analysis of sense/antisense correlation.
(A) 1669 gene loci identified with overlapping longest non-coding transcript. Additional location details (chromosome, strand, start and end coordinates) for the gene and overlapping non-coding transcripts are provided.
(B) Data description – Additional details (name, description, methods for differential expression and correlation analyses) for data used in this study are provided.
